# Species Composition and Trichothecene Genotype Profiling of *Fusarium* Field Isolates Recovered from Wheat in Poland

**DOI:** 10.3390/toxins10080325

**Published:** 2018-08-10

**Authors:** Katarzyna Bilska, Sebastian Jurczak, Tomasz Kulik, Ewa Ropelewska, Jacek Olszewski, Maciej Żelechowski, Piotr Zapotoczny

**Affiliations:** 1 Department of Botany and Nature Protection, University of Warmia and Mazury in Olsztyn, Plac Łódzki 1, 10-727 Olsztyn, Poland; sebastian.jurczak@uwm.edu.pl (S.J.); tomaszkulik76@gmail.com (T.K.); maciej.zelechowski92@gmail.com (M.Ż.); 2 Department of Systems Engineering, Faculty of Engineering, University of Warmia and Mazury in Olsztyn, Heweliusza 14, 10-718 Olsztyn, Poland; ewa.ropelewska@uwm.edu.pl (E.R.); zap@uwm.edu.pl (P.Z.); 3 Experimental Education Unit, Oczapowskiego 8, 10-719 Olsztyn, Poland; jacolsz@uwm.edu.pl

**Keywords:** *Fusarium*, Fusarium head blight, trichothecenes, genotypes, *Tri* core cluster, qPCR, wheat

## Abstract

Fusarium head blight (FHB) of cereals is the major head disease negatively affecting grain production worldwide. In 2016 and 2017, serious outbreaks of FHB occurred in wheat crops in Poland. In this study, we characterized the diversity of Fusaria responsible for these epidemics using TaqMan assays. From a panel of 463 field isolates collected from wheat, four *Fusarium* species were identified. The predominant species were *F. graminearum* s.s. (81%) and, to a lesser extent, *F. avenaceum* (15%). The emergence of the 15ADON genotype was found ranging from 83% to 87% of the total trichothecene genotypes isolated in 2016 and 2017, respectively. Our results indicate two dramatic shifts within fungal field populations in Poland. The first shift is associated with the displacement of *F. culmorum* by *F. graminearum* s.s. The second shift resulted from a loss of nivalenol genotypes. We suggest that an emerging prevalence of *F. graminearum* s.s. may be linked to boosted maize production, which has increased substantially over the last decade in Poland. To detect variation within *Tri* core clusters, we compared sequence data from randomly selected field isolates with a panel of strains from geographically diverse origins. We found that the newly emerged 15ADON genotypes do not exhibit a specific pattern of polymorphism enabling their clear differentiation from the other European strains.

## 1. Introduction

**Figure 1:**
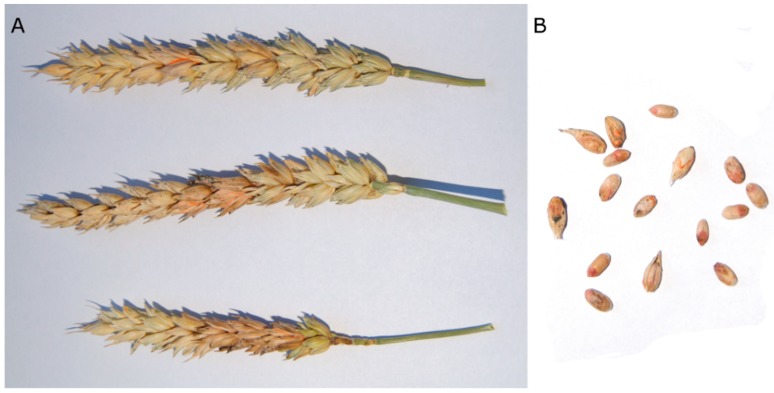
Fusarium head blight of wheat. (**A**) symptomatic heads. (**B**) *Fusarium*-damaged kernels.

Fusarium head blight (FHB, syn. scab) is one of the most devastating small-grain cereal diseases worldwide. Although globally FHB mostly affects barley, rye, oats, and triticale, it is considered the most precarious head disease of wheat [1]. Warm and humid weather conditions at the flowering stage are conducive to disease development [2,3,4]. Symptoms may occur over the entire head or on just a few spikelets, which leads to the formation of *Fusarium*-damaged kernels (FDK) (Figure 1). Accumulation of various mycotoxins in the grain affected by FHB pose a significant risk to food and feed safety [5].

Control of the disease is focused on agricultural practices, fungicides, and cultivar resistance [6]. Among fungicides, azoles play a major role in controlling FHB. However, the increased application of azoles resulted in resistant strains recovered from *F. graminearum* field populations [7,8,9]. The development of resistant varieties appears to be the most promising approach for managing the disease. However, improvement of resistance to FHB is a continuous challenge because of environment effects, genotype-by-environment interactions [10], and a broad range of *Fusarium* species associated with FHB.

*F. graminearum* sensu lato (s.l.) is nowadays the most frequently isolated causal agent of FHB worldwide. Phylogenetic species recognition, with genealogical concordance [11], has provided strong genetic evidence that *F. graminearum* sensu lato comprises at least 16 biogeographically structured and phylogenetically distinct species, also known as the *Fusarium graminearum* species complex (FGSC) [12,13,14,15]. *F. graminearum* sensu stricto (s.s.) is the dominant FGSC species associated with FHB in North and South America [16,17,18,19], whereas *F. asiaticum* appears to be the major species in temperate regions of Asia [15,20,21,22]. In Europe, *F. graminearum* s.s. displaced *F. culmorum*, which had been the major FHB agent of wheat since the 1840s [23]. However, the predomination of *F. culmorum* in some European localities identified in several surveys may be explained by the influence of climatic conditions in increasing the prevalence of each species [24].

FHB epidemics occurred in Poland in 1974 and 1999, and over the next decade, the incidence of the disease in small grain cereals was rather limited [25,26,27,28]. Despite regional differences in pathogen composition, previous Polish surveys highlighted the general predominance of three species: *F. culmorum; F. graminearum* s.l.; and, to a lesser extent, *F. avenaceum* [28,29,30]. Stępień et al. [28] indicated that predominance of *F. graminearum* is associated with displacement of *F. culmorum* by *F. graminearum*, which corresponds to previous surveys from the Netherlands [31] and Luxembourg [32]. However, the suggested shift was not confirmed in further surveys of Wiśniewska et al. [29] by incorporating a much larger panel of field isolates. In the 2009 growing season, FHB outbreaks were reported in both northern and southern Poland [29,33]. A large-scale survey by Chełkowski et al. [33] showed that *F. graminearum* s.l. and *F. culmorum* were responsible for the last outbreak, with emphasis on the former.

Both *F. culmorum* and *F. graminearum* s.s. can belong to different chemotypes: 3ADON (producing DON and 3ADON), 15ADON (producing DON and 15ADON) (absent in *F. culmorum*), and NIV (producing NIV and 4ANIV) [34]. Knowledge of chemotype composition within *Fusarium* field populations has been found to be important in determining population structure, monitoring chemotype shifts, and predicting the presence of trichothecene compounds in grains [22,35,36]. Trichothecene chemotypes appear to differ in pathogenicity [16,37,38], susceptibility to fungicides [39], and may differ in the total amount of toxin produced. For example, the 3ADON chemotype poses a greater risk to food safety than 15ADON, as it was found to produce more DON [16,40,41].

Field populations of trichothecene producers are often characterized by incorporation of mycotoxin genotyping tools allowing rapid detection of 3, 15ADON, and NIV genotypes [42,43,44,45]. Over the last 15 years, shifts between *Tri* genotypes/chemotypes have been observed in geographically diverse locations such as North America, China, Denmark, and Luxembourg [15,16,22,37,44,46,47].

The important areas in which to focus research on *Fusarium* species are the natural boundaries and climates of agricultural regions, which may define pathogen potential [6]. The climate in Poland is temperate, with relatively cold winters and warm summers, where northern maritime air from the Atlantic and eastern continental air often collide, causing high daily and annual variability in the weather patterns. This high climate variability may affect pathogen profiles that may differ from fungal populations found in the more humid climate of western Europe. As mentioned earlier, previous data on species composition responsible for FHB epidemics in Poland comes from relatively old surveys [28,29,33]. In addition, there are no current data on the population subdivision of Fusaria associated with *Tri* genotype differences. There is an urgent need to update results, mainly from the most recent FHB outbreaks, which occurred during the 2016 and 2017 growing seasons. In 2016, the disease occurred over the entire country, while in 2017, FHB occurrence was restricted to northern Poland. We have characterized the collection of field isolates responsible for FHB in Poland in both growing seasons. The isolates were recovered mainly from FDK of wheat from geographically diverse fields. To determine *Fusarium* species and trichothecene genotypes, we used different TaqMan-based assays. To detect variation within *Tri* core clusters, we compared sequence data from randomly selected field isolates with a panel of strains from geographically diverse origins. The updated results of this study could be used to develop a reliable plant protection strategy against currently emerged fungal species.

## 2. Results

### 2.1. Determination of Fungal Species and Trichothecene Genotypes

From the panel of 463 field isolates collected from wheat, four *Fusarium* species were identified (Table 1). The predominant species were *F. graminearum* s.s. (81.2%) and, to a lesser extent, *F. avenaceum* (14.7%). Both *F. culmorum* and *F. poae* were occasionally isolated. Our results indicated high regional and temporal differences in species composition. For example, predomination of *F. graminearum* s.s. appeared to be complete in wheat samples from northern Poland (Łajsy, Tywęzy, Tczew and Malbork), where *F. graminearum* s.s. was the solely isolated species. In contrast, other sampling locations (Kętrzyn, Ostrołęka, and Warsaw West Counties, 2017) exhibited a slightly decreased incidence of *F. graminearum* s.s., ranging from 46.1% to 54.6% of the total FDKs analyzed. Barley samples analyzed in this study showed the lowest (<17%) incidence of *F. graminearum* s.s. (Table 1).

In 2016, where FHB affected an entire country, *F. graminearum* s.s. accounted for 91% of the diseased wheat kernels. In 2017, the amount of *F. graminearum* s.s. isolates recovered from FDKs decreased to 68% and was restricted to northern Poland. Dramatic regional differences were also found when analyzing *F. avenaceum* distribution. In general, the incidence of *F. avenaceum* on wheat ranged from 5.3% in 2016 to 27.4% in 2017. However, in three locations (Kętrzyn County, Ostrołęka County, and Warsaw West County), *F. avenaceum* accounted for over 44% of the diseased kernels. Moreover, an unexpectedly high incidence of *F. avenaceum* was found on barley kernels from Modzurów, accounting for 80.4% of FDKs. *F. avenaceum* was not recovered from 5 out of 17 wheat sampling locations.

In contrast to previous surveys by Wiśniewska et al. [29] and Chełkowski et al. [33], a surprisingly low incidence of *F. culmorum* was found in our study (3.9%). Only one isolate was recognized as *F. poae* (0.2%). *F. culmorum* was identified in 4 out of 17 locations. None of the *F. poae* isolates were found in 2017.

Genotyping of a collection of 394 *F. culmorum*/*F. graminearum* s.s. isolates from wheat kernels showed a high prevalence of the 15ADON genotype in Poland. In the 2016 and 2017 growing seasons, the 15ADON genotype accounted for 82.9% and 87.4% of *F. culmorum*/F. graminearum s.s. isolates, respectively. All 333 isolates identified as 15ADON genotype belonged to *F. graminearum* s.s. Among the total of 61 3ADON genotypes, 70.5% and 29.5% belonged to *F. graminearum* and *F. culmorum*, respectively. The 3ADON genotype was more prevalent in 2016, accounting for 17.1% of the total *Tri* genotypes. In 2017, a shift towards the 15ADON genotype was observed, leading to a decreased incidence of 3ADON genotypes (12.6%). None of the NIV genotype was determined in our collection of field isolates.

### 2.2. Comparative Analysis of the Tri Core Cluster

In this study, we asked if an additional level of diversity within core *Tri* clusters enables specific characterization of the emerged 15ADON genotypes. To achieve it, the complete *Tri* core clusters from four emerging (isolated in 2016) 15ADON field isolates were compared with the panel of 31 strains from geographically diverse origins. Our collection also included four historical Polish isolates recovered between 2003 and 2004 (Table 2).

Sequence data comparison did not detect gene translocations or gene gain and loss polymorphisms. SNPs (single nucleotide polymorphisms) were the most common type of DNA polymorphism determining genetic variability of the strains within this cluster. All identified SNPs were synonymous.

The emerging 15ADON isolates did not show a specific pattern of polymorphism enabling their clear differentiation from the other European strains. However, we showed that the studied strains displayed polymorphism related to their European and American origins. The most remarkable differences between these two groups were found within the 1333 bp end of the *Tri* core cluster totaling 55 SNPs (Supplementary Figure S1). Thirty-one SNPs were located within the *Tri*14 gene (1170 bp), while the remaining 24 SNPs were found within the short (163 bp) upstream region of the *Tri*14 gene.

## 3. Discussion

FHB surveys performed in Europe over the last few decades have verified an increased predominance of *F. graminearum* sensu lato in different European countries, for example, The Netherlands [31], France [48,49], Germany [50], and Italy [51,52].

A previous large-scale survey by Chełkowski et al. [33] highlighted the increasing predomination of *F. graminearum* s.l. in wheat in Poland during the FHB epidemics in 2009. At the same time, however, considerable incidence of *F. culmorum* was found in some Polish localities [29,33]. In our study, we showed that *F. graminearum* s.s. dramatically predominated in the growing seasons of both 2016 and 2017. Our results highlighted, for the first time, a huge disproportion between *F. graminearum* s.s. and *F. culmorum*. Although some regional and temporal differences were found, it is worth mentioning that these changes were mainly attributed to a noticeable shift towards *F. avenaceum* in 2017.

Our results showed that the 15ADON genotype predominated in diseased kernels, ranging from 83% to 87% of the total trichothecene genotypes isolated in 2016 and 2017, respectively. The revealed predomination of the 15ADON genotype in Poland is in line with other European surveys [47,49,50,51,52,53], however, 3ADON genotypes [54] and NIV [55] genotypes have also been observed. Surprisingly, none of the NIV genotypes have been identified within either *F. culmorum* or *F. graminearum* s.s.

The emergence of *F. graminearum* in Europe has been mainly linked to boosted production of maize as the primary host of *F. graminearum* [23,31,56]. Maize production results in large amounts of residues, which promote the production of ascospores [57], which appear to greatly outnumber airborne conidia [58] and might be carried over long distances [59]. Recently, it was found that 15ADON isolates have an advantage in perithecia formation and ascospore release [60]. The observed dramatic emergence of *F. graminearum* s.s. in Poland may also be linked to the rise in maize production. Since the last reports on FHB incidence in 2009 [29,33], the maize cultivation area increased by nearly 42%, averaging 595,000 ha in 2016 (http://www.fao.org/faostat/en/#data/QC), which is close to the maize cultivation area in Germany (416,000 ha) and Italy (660,000 ha). In both countries, as well as in France (the largest maize producer in Europe), predomination of the 15ADON genotype of *F. graminearum* has been highlighted [49,50,51].

Besides cropping systems, changes in climate patterns, such as the increase in annual temperature, have also been suggested as key factors affecting recent emergence of *F. graminearum* s.s. [31,56,61,62,63]. Indeed, since 1951, reports from Poland have highlighted the increase in the annual mean temperature by 1.1–2.2 °C [64]. The average global temperature for the 2013–2017 period was 1.0 °C above pre-industrial levels and was the highest five-year average on record [65].

Production of different analogs of trichothecene compounds by fungi affect their virulence [16,37,41] and distribution [66,67]. The regional and temporal differences in *Tri* genotype frequency may change over time and space. In addition, a chemotype occupying a certain geographic location can be replaced by another. Most recent chemotype shifts appear to result from the introduction of new genotypes into new areas [23]. Such examples include 3ADON genotypes of *F. graminearum* s.s. in Canada [16], 3ADON genotypes of *F. asiaticum* in Southern China [22], and the NIV genotype of *F. asiaticum* in the southern United States [68] and Brazil [69].

Kelly and Ward [66] showed that among the number of genomic regions, *Tri* genes exhibit the strongest signals of selection contributing to population divergence. In this study, we found that the newly emerged Polish 15ADON genotypes do not exhibit specific patterns of polymorphism enabling their clear differentiation from the other European strains. This may suggest previously uncovered evidence of frequent gene flow between different European populations of *F. graminearum* s.s. [50]. However, we showed that the strains display a pattern of polymorphism within the *Tri* core cluster regarding their European and American origins. The highest number of SNPs was reported in the *Tri*14 gene (Supplementary Figure S1). This gene might be required for high virulence of *F. graminearum* and seems to regulate trichothecene biosynthesis in planta [70]. Even though all SNPs were synonymous, this adaptation might provide better competitiveness in a new environment. The latest evidence from comparative and experimental studies indicates that synonymous mutations may not be selectively neutral [71,72,73,74,75,76]. The revealed geographic pattern of polymorphism highlights hallmarks of divergent evolutionary history of *F. graminearum* s.s. that reflects barriers in transcontinental introductions. However, we suggest that our finding has been validated with only relatively few strains/isolates, so confirmation is critical with more representative isolates in the future. As stated by Kelly and Ward [66], the localized gene flow or potential transcontinental introductions of novel genotypes into new locations could pose further challenges to FHB management, because selection drives the emergence of more pathogenic fungi. In this regard, international biosecurity regulatory mechanisms incorporating molecular data will be critical to minimize their spread.

## 4. Materials and Methods

### 4.1. Fusarium-Damaged Kernels (FDKs)

**Figure 2:**
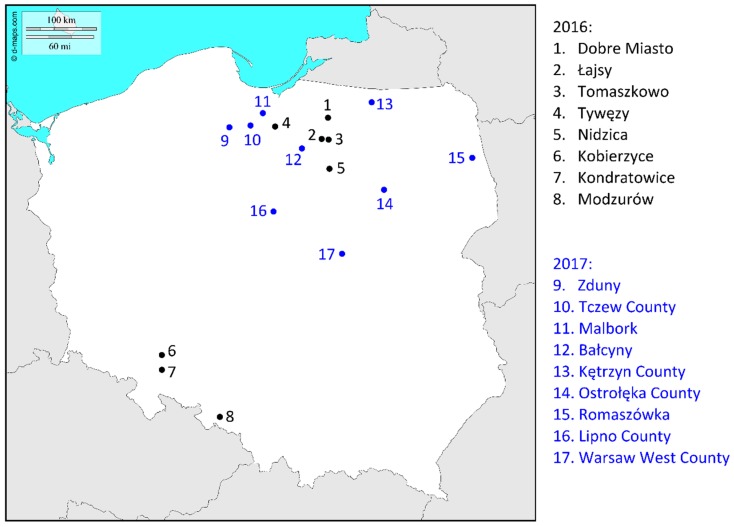
Locations of fields from which *Fusarium*-damaged kernels (FDKs) were collected for analyses.

Kernels with visible FHB symptoms were manually sampled from different wheat fields located in 17 different locations (Figure 2). Additionally, one population of diseased barley kernels from Modzurów were also included. Kernels were plated on potato dextrose agar (PDA) in Petri dishes and incubated at room temperature in darkness. After 4–6 days of incubation, *Fusarium*-like colonies were transferred to new PDA plates for further analysis. *Fusarium-*like colonies were selected based on visual observation of fungal growth on PDA. Fusaria grow on PDA rapidly producing abundant, dense, white, and aerial mycelium. They can produce various pigments, with colors ranging from white, through pink, salmon-pink and carmine red, to purple [77].

For storage purposes, colonies were transferred to new PDA plates, and after six days of culturing, mycelia were covered with 1.5 g of sterile soil [78]. All plates were held at RT for 7–10 days until the soil was overgrown by mycelium. A total of 519 *Fusarium* isolates (463 from wheat kernels and 56 from barley kernels) were assigned with unique isolate codes and were stored at −25 °C in the fungal collection of the Department of Botany and Nature Protection, University of Warmia and Mazury in Olsztyn, Poland.

### 4.2. Preparation of Cell Lysates for qPCR

A patch of mycelium (approximately 0.1–0.2 mg) was scraped from the surface of the PDA plate and transferred to tubes with 1 mm silica spheres (Lysing matrix C, MP Biomedicals, Santa Ana, CA, USA). Samples were homogenized at 40 s at the speed 6.0 m/s in 1 mL of deionized water on a FastPrep-24 instrument (MP Biomedicals, Santa Ana, CA, USA). After homogenization, 40 µL of homogenate was subsequently diluted with 160 µL of water and incubated for 2 min at 90 °C. 2 µL of aqueous phase was used for qPCR.

### 4.3. Determination of Fungal Species and Tri Genotypes

Four species-specific TaqMan assays were used to identify all field isolates to the species level (Table 3). Trichothecene genotypes were determined using TaqMan assays targeting the *Tri*12 gene [43]. Each reaction was carried out in three replicates.

### 4.4. DNA Sequencing, Assembly, and Annotation of Tri Core Clusters

For next generation sequencing (NGS), DNA from 35 isolates/strains was extracted from 0.1 g of mycelium as previously described in Kulik et al. [82]. A patch of mycelium was scraped from the surface of the PDA plate and homogenized in tubes containing 1 mm silica spheres (Lysing matrix C, MP Biomedicals, Santa Ana, CA, USA) on a FastPrep-24 instrument (MP Biomedicals, Santa Ana, CA, USA). Total genomic DNA was extracted with the use of the Quick-DNA Plant/Seed Miniprep Kit (Zymo Research, Irvine, CA, USA) according to the manufacturer’s protocol.

Whole-genome sequencing was performed at Macrogen (Seoul, South Korea). Genome libraries were constructed using a TruSeq DNA PCR-free library preparation kit (Illumina, San Diego, CA, USA). An Illumina HiSeq X platform was used to sequence the genomes using a paired-end read length of 2 × 150 bp with an insert size of 350 bp. For assembling, de-multiplexed and trimmed reads were aligned to the complete sequence of the core *Tri* clusters deposited in the GenBank database under accession numbers: KU572424 (3ADON genotype) and KU572428 (15ADON genotype) [83]. Sequence reads were aligned with Geneious (v.6.1.6 created by Biomatters, Auckland, New Zealand, available from http://www.geneious.com), as previously described in Kulik et al. [82]. Annotations were performed using Geneious software based on sequence data deposited under accession numbers KU572424 and KU572428. Complete core *Tri* clusters have been deposited in the NCBI database under the GenBank accession numbers MH514924-MH514957. The identity of all 35 analyzed strains were confirmed using *TEF*1 gene (translation elongation factor). The sequences have been deposited in GeneBank under accession numbers MH572233-MH572267.

## Figures and Tables

**Table 1:** TaqMan-based identification of the *Fusarium* species and their chemotypes in wheat and barley kernels in Poland.

Year	Localization	Number of Isolates	*F. graminearum* s.s.			*F. culmorum*		*F. avenaceum*	*F. poae*
			Total	15ADON	3ADON	Total	3ADON		
	Wheat kernels								
**2016**	Dobre Miasto	22	21 (95.45%)	15 (71.43%)	6 (28.57%)	0	0	1 (4.55%)	0
	Łajsy	85	85 (100%)	75 (88.24%	10 (11.76%)	0	0	0	0
	Tomaszkowo	57	54 (94.74%)	45 (83.33%)	9 (16.67%)	1 (1.75%)	1 (100%)	2 (3.51%)	0
	Tywęzy	5	5 (100%)	5 (100)	0	0	0	0	0
	Nidzica	20	17 (85%)	15 (88.24%)	2 (11.76%)	0	0	3 (15%)	0
	Kobierzyce	7	6 (85.71%)	6 (100%)	0	0	0	1 (14.29)	0
	Kondratowice	11	10 (90.91%)	10 (100%)	0	0	0	0	1 (9.09%)
	Modzurów	59	44 (74.58%)	37 (84.09%)	7 (15.91%)	8 (13.56%)	8 (100%)	7 (11.86%)	0
	**Total in 2016**	**266**	**242 (90.98%)**	**208 (85.95%)**	**34 (14.05%)**	**9 (3.38%)**	**9 (100%)**	**14 (5.26%)**	**1 (0.38%)**
**2017**	Zduny	11	9 (81.82%)	8 (88.89%)	1 (11.11%)	0	0	2 (18.18%)	0
	Tczew County	26	26 (100%)	25 (96.15%)	1 (3.85%)	0	0	0	0
	Malbork	6	6 (100%)	6 (100%)	0	0	0	0	0
	Bałcyny	8	5 (62.50%)	5 (100%)	0	0	0	3 (37.50%)	0
	Kętrzyn County	11	6 (54.55%)	5 (83.33%)	1 (16.67%)	0	0	5 (45.45%)	0
	Ostrołęka County	76	35 (46.05%)	31 (88.57%)	4 (11.43%)	7 (9.21%)	7 (100%)	34 (44.74%)	0
	Romaszówka	15	9 (60.00%)	7 (77.78%)	2 (22.22%)	2 (13.33%)	2 (100%)	4 (26.67%)	0
	Lipno County	33	32 (96.97%)	32 (100%)	0	0	0	1 (3.03%)	0
	Warsaw West County	11	6 (54.55%)	6 (100%)	0	0	0	5 (45.45%)	0
	**Total in 2017**	**197**	**134 (68.02%)**	**125 (93.28%)**	**9 (6.72%)**	**9 (4.75)**	**9 (100%)**	**54 (27.41%)**	**0**
**Total wheat kernels**		**463**	**376 (81.21%)**	**333 (88.56%)**	**43 (11.44%)**	**18 (3.89%)**	**18 (100%)**	**68 (14.69%)**	**1 (0.22%)**
Barley kernels from Modzurów		56	9 (16.07%)	9 (100%)	0	0	0	45 (80.36%)	2 (3.57%)

**Table 2:** List of *F. graminearum* s.s. 15ADON strains used for comparison of complete core *Tri* clusters.

Strain	Origin	Host	Year of Isolation	Culture Collection
16-21-z	Poland, Dobre Miasto	winter wheat	2016	1
16-92-z	Poland, Modzurów	winter wheat	2016	1
16-43-tp	Poland, Tomaszkowo	winter wheat	2016	1
16-462-z	Poland, Modzurów	winter wheat	2016	1
03132	Poland, Lewin Brzeski	winter wheat	2003	1
04286	Poland, Bałcyny	winter wheat	2004	1
04501	Poland, Martąg	winter wheat	2004	1
CBS 138561	Poland	wheat	2010	4
37	Germany	unknown	1994	2
74b	Germany	unknown	2004	2
N2-1	Germany, Uelzen	winter wheat	2017	1
N4-1	Germany, Uelzen	winter wheat	2017	1
N4-2	Germany, Uelzen	winter wheat	2017	1
N5-1	Germany, Uelzen	winter wheat	2017	1
N6-1	Germany, Uelzen	winter wheat	2017	1
N6-2	Germany, Uelzen	winter wheat	2017	1
N7-1	Germany, Uelzen	winter wheat	2017	1
N8-2	Germany, Uelzen	winter wheat	2017	1
N10-1	Germany, Uelzen	winter wheat	2017	1
N10-2	Germany, Uelzen	winter wheat	2017	1
237	Luxembourg	winter wheat	2007	2
321	Luxembourg	winter wheat	2007	2
630	Luxembourg	winter wheat	2007	2
09-03a	Luxembourg	winter wheat	2009	2
09-04a	Luxembourg	winter wheat	2009	2
09-5a	Luxembourg	winter wheat	2009	2
09-13a	Luxembourg	winter wheat	2009	2
09-21a	Luxembourg	winter wheat	2009	2
09-53b	Luxembourg	winter wheat	2009	2
St-6	Russia, Stavropol region	winter wheat	2015	3
St-9	Russia, Stavropol region	winter wheat	2014	3
Kr 275-1	Russia, Krasnodar region	winter wheat	2014	3
433-2	Russia, Kabardino-Balkaria	winter wheat	2014	3
CBS 134070	USA, Illinois, Urbana	Miscanthus giganteus	unknown	4
GZ3639, CBS 110266	USA, Kansas	wheat	unknown	4
CBS 139513	Argentina, Tandil	barley	2011	4
CBS 139514	Argentina, Tapalqué	barley	2010	4
114-2	Argentina, Loberia	barley	2012	1
23-4	Argentina, Lauquen	barley	2011	1

1—Fungal Culture Collection of the Department of Botany and Nature Protection of the University of Warmia and Mazury in Olsztyn, Poland; 2—Fungal Culture Collection of the Department of Environmental Research and Innovation of the Luxembourg Institute of Science and Technology, Luxembourg; 3—All-Russian Institute of Phytopathology, Russia; 4—Westerdijk Fungal Biodiversity Institute, The Netherlands.

**Table 3:** List of TaqMan assays used to determine species and trichothecene genotypes.

	Primer/Probe Sequence	Reaction Reagents	Reaction Condition	References
**Species**				
*F. culmorum*	F: TCGTTGACGGTGAGGGTTGT R: GACTCGAACACGTCAACCAACTT Probe: FAM-CGGTTATTATTTCGAAAAGT- MGB	2 μL gDNA, 14.3 μL H_2_O, 6.7 μM of each primer, 1.7 μM of probe, 3.6 μL TaqMan Fast Advanced Master Mix (Applied Biosystems, Foster City, CA, USA).	95 °C for 20 s, (95 °C for 1 s, 60 °C for 30 s) × 40	[79]
*F. graminearum* s.s.	F: TGGCCTGAATGAAGGATTTCTAG R: CATCGTTGTTAACTTATTGGAGATG Probe: FAM-TTAAACACTCAAACACTACA- MGB			[80]
*F. avenaceum*	F: CCATCGCCGTGGCTTTC R: CAAGCCCACAGACACGTTGT Probe: FAM-ACGCAATTGACTATTGC-MGB	2 μL gDNA, 10.8 μL H_2_O, 6.7 μM of each primer, 1.7 μM of probe, 7.2 μL TaqMan Fast Advanced Master Mix (Applied Biosystems, Foster City, CA, USA).	95 °C for 20 s, (95 °C for 1 s, 60 °C for 50 s) × 40	[81]
*F. poae*	F: AAATCGGCGTATAGGGTTGAGATA R: GCTCACACAGAGTAACCGAAACCT Probe: FAM-CAAAATCACCCAACCGACCCTTTC-TAMRA		50 °C for 2 min, 95 °C for 10 min, (95 °C for 15 s, 60 °C for 60 s) × 40	
***Tri* genotype**				
3ADON	F: CATGCGGGACTTTGATCGAT R: TTTGTCCGCTTTCTTTCTATCATAAA Probe: FAM-CTCACCGATCATGTTC-MGB	2 μL gDNA, 10.8 μL H_2_O, 6.7 μM of each primer, 1.7 μM of probe, 7.2 μL TaqMan Fast Advanced Master Mix (Applied Biosystems, Foster City, CA, USA).	95 °C for 20 s, (95 °C for 1 s, 60 °C for 50 s) × 40	[43]
15ADON	F: TCCAATCATTGCCAGCCTCTA R: TGATGCGGAACATGGTCTGT Probe: FAM-ATGAGGGACTTTGACCAAT-MGB			
NIV	F: TCGCCAGTCTCTGCATGAAG R: CCTTATCCGCTTTCTTTCTATCATAAA Probe: FAM-CTGATCATGTCCCGCATC-MGB			

## Supplementary Materials

The following are available online at http://www.mdpi.com/2072-6651/10/8/325/s1, Figure S1: Comparison of complete sequences of the *Tri*14 gene of *F. graminearum* s.s. 15ADON strains from geographically diverse origins. Sequences highlighted with grey indicate the *Tri*14 gene.
