# Supplementary figures and images for: Species Composition and Trichothecene Genotype Profiling of Fusarium Field Isolates Recovered from Wheat in Poland
Source: Toxins (Basel). 2018 Aug 10;10(8):325. doi: 10.3390/toxins10080325 (PMC6115980; doi:10.3390/toxins10080325)

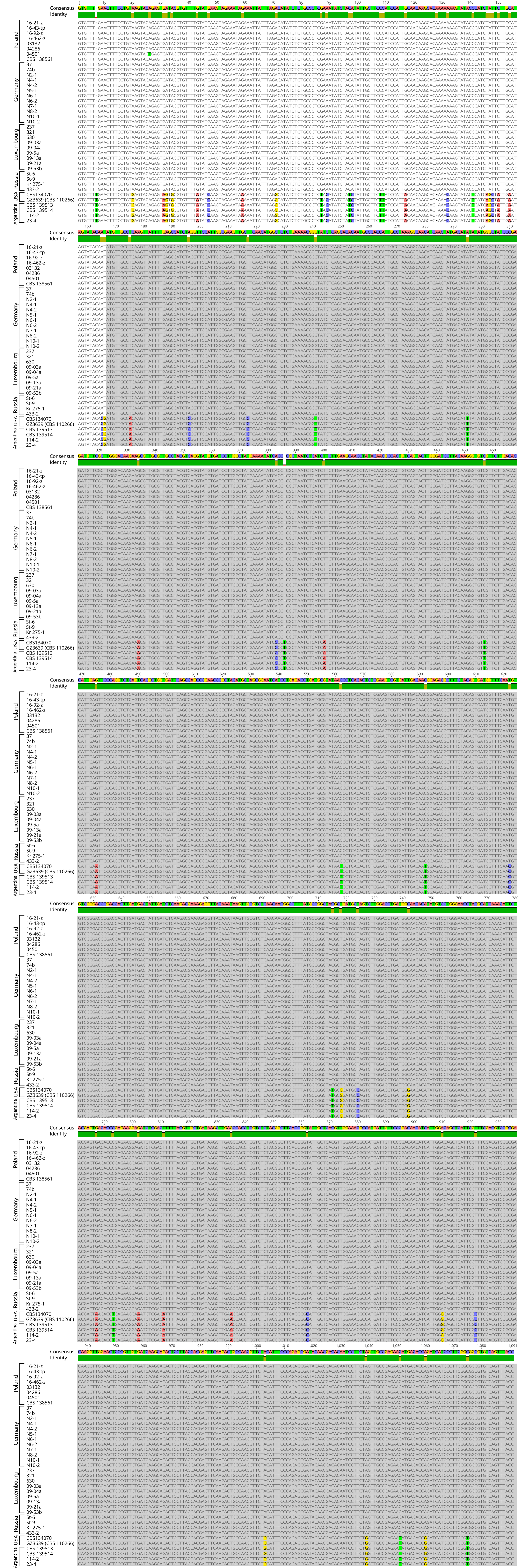

Supplement: Supplementary file 1 [file toxins-10-00325-s001.pdf]
